# Typhoid Fever Cured in Its First Stage

**Published:** 1892-05

**Authors:** J. C. White

**Affiliations:** Portchester, N. Y.


### TYPHOID FEVER CURED IN ITS FIRST STAGE.

J. C. White, M. D., Portchester, N. Y.

Cases from Practice.

W. E., age fifteen, student.—Was called to see him on Dec. 28th, A. M.; found his pulse 110, temperature 102; some nervous trembling. The mother said he had been somewhat delirious during the night, seeing and talking to people not present. She had a family case of remedies, and had given him Acon. during the night, and had just prescribed Hyos. As his delirium was mild and of the good-natured tone, I continued the prescription.

December 29th, A. M.—Patient had not rested or slept at all; delirium increased; temperature 102, pulse 112. On ascertaining that he had suffered exposure, had his feet wet, etc., I gave him Rhus$^{cc}$, a dose every three hours, and called in the evening. No rest; delirium increased, constant moaning, jactitation of muscles, temperature 104, pulse 120. Continued Rhus every half-hour for six times, then three hours apart.

December 30th, A. M.—Patient had no rest; constant delirium; "was on the school-ship St. Mary, bombarding Valparaiso; the North Pole, and other places;" sordes on teeth, tongue dry, heavily coated, bowels constipated, tympanitic; temperature 103, pulse 120; Bry.$^{cc.}$ every half-hour until symptoms better, then every three hours. In the evening delirium was better, had slept a little; temperature 103, pulse 118. Continued Bry. every three hours, intermediate doses to be given if more restless in the night.

December 31st, A. M.—Patient greeted me with the usual salutation; had slept most of the night, very little delirium, and none of the usual moaning so characteristic of the remedy; temperature 101, pulse 100. Continued Bry. The bowels moved naturally next day and convalescence established.

January 6th.—Was sitting up in bed and took meals in that position; on the 10th was around the house. The regular rise and fall of temperature during the first three days of my attendance, the slightly tympanitic abdomen and the tenderness over right iliac region, establishes the diagnosis of typhoid fever. There was no diarrhœa or ulceration of Peyer's glands. The happy termination of the case is sufficient, it seems to me, to convince the most incredulous that typhoid may be cured in its first stage.

